# Purpura fulminans with Lemierre’s syndrome caused by *Gemella bergeri* and *Eikenella corrodens*: a case report

**DOI:** 10.1186/s12879-018-3437-6

**Published:** 2018-10-19

**Authors:** Toshinobu Yamagishi, Mayu Hikone, Kazuhiro Sugiyama, Takahiro Tanabe, Yasuhiro Wada, Michiko Furugaito, Yuko Arai, Yutaka Uzawa, Ryo Mizushima, Keisuke Kamada, Yasutomo Itakura, Shigekazu Iguchi, Atsushi Yoshida, Ken Kikuchi, Yuichi Hamabe

**Affiliations:** 1 Tertiary Emergency Medical Center, Tokyo Metropolitan Bokutoh Hospital, 4-23-15 Kotobashi, Sumida-ku, Tokyo, 130-8575 Japan; 2 Department of Infectious Diseases, Tokyo Women’s Medical University, 8-1 Kawada-cho, Shinjuku-ku, Tokyo, 162-8666 Japan

**Keywords:** *Eikenella corrodens*, *Gemella bergeri*, Lemierre’s syndrome, MALDI-TOF MS, Purpura fulminans, Septic shock, 16S rRNA gene

## Abstract

**Background:**

*Gemella bergeri* is one of the nine species of the genus *Gemella* and is relatively difficult to identify. We herein describe the first case of septic shock due to a *Gemella bergeri* coinfection with *Eikenella corrodens*.

**Case presentation:**

A 44-year-old Asian man with a medical history of IgG4-related ophthalmic disease who was prescribed corticosteroids (prednisolone) presented to our hospital with dyspnea. On arrival, he was in shock, and a purpuric eruption was noted on both legs. Contrast enhanced computed tomography showed fluid retention at the right maxillary sinus, left lung ground glass opacity, and bilateral lung irregular opacities without cavitation. Owing to suspected septic shock, fluid resuscitation and a high dose of vasopressors were started. In addition, meropenem, clindamycin, and vancomycin were administered. Repeat computed tomography confirmed left internal jugular and vertebral vein thrombosis. Following this, the patient was diagnosed with Lemierre’s syndrome. Furthermore, he went into shock again on day 6 of hospitalization. Additional soft tissue infections were suspected; therefore, bilateral below the knee amputations were performed for source control. Cultures of the exudates from skin lesions and histopathological samples did not identify any pathogens, and histopathological findings showed arterial thrombosis; therefore it was concluded that the second time shock was associated with purpura fulminans. Following this, his general status improved. He was transferred to another hospital for rehabilitation. The blood culture isolates were identified as *Gemella bergeri* and *Eikenella corrodens*. *Gemella bergeri* was identified by matrix-assisted laser desorption ionization-time of flight mass spectrometry and confirmed by 16S rRNA gene sequencing later. The primary focus of the infection was thought to be in the right maxillary sinus, because the resolution of the fluid retention was confirmed by repeat computed tomography.

**Conclusions:**

*Gemella bergeri* can be the causative pathogen of septic shock. If this pathogen cannot be identified manually or through commercial phenotypic methods, 16S rRNA gene sequencing should be considered.

## Background

*Gemella bergeri* is one of nine species (the others include *G. haemolysans*, *G. morbillorum*, *G. sanguinis*, *G. asaccharolytica*, *G. taiwanensis*, *G. parahaemolysans*, *G. cuniculi,* and *G. palanticanis*) belonging to the genus *Gemella* [1–3]. Because conventional biochemical methods may result in misidentification of *Gemella* as viridans group streptococci or other related organisms, they are relatively difficult to identify and considered uncommon organisms [4]. *G. bergeri* belongs to the normal flora of the oral cavity, and digestive and urinary tract, and was isolated for the first time by Collins et al. in 1998 [5]. Since then, it is gradually being recognized and so far, 13 cases have been reported [4–11] (Table 1). Except for one case associated with cardiogenic shock due to perforation of the mitral valve [4], there are no cases of septic shock due to *G. bergeri* infection.

**Table 1:** Cases of *Gemella berigeri* from 1998 to 2018

Case	Age	Gender	Disease	Underlying disease	Antibiotics	Reson for changing antibiotics	Blood culture	How to diagnose	Operation	Outcome	Reference
1	42	Male	I ▪ E	Mitral valve prolapse, periodontitis	NA	NA	NA	16S rRNA gene sequence	Valve replacement	Admission	[5]
2	NA	NA	I・E	NA	NA	NA	NA	16S rRNA gene sequence	NA	Survived	[5]
3	NA	NA	I・E	NA	NA	NA	NA	16S rRNA gene sequence	NA	Survived	[5]
4^a^	NA	NA	NA	NA	NA	NA	NA	16S rRNA gene sequence	NA	Survived	[5]
5^a^	NA	NA	NA	NA	NA	NA	NA	16S rRNA gene sequence	NA	Survived	[5]
6^a^	NA	NA	NA	NA	NA	NA	NA	16S rRNA gene sequence	NA	Survived	[5]
7	32	Male	I・E	Bicuspid aortic valve	Ampicillin, gentamicin, refampicin	No	Gram-positive cocci	16S rRNA gene sequence	Ring abscess curettage, valve replacement	Survived	[6]
8	15	Male	I・E	Tetralogy of Fallot, pulmonary atresia	Vancomycin, gentamicin →ceftriaxone, refampicin	Side effect	Gram-positive cocci	16S rRNA gene sequence	No	Survived	[7]
9	24	Male	I・E	Rheumatic heart disease	Ceftriaxone, gentamicin	No	Gram-positive cocci	Phenotypic method	Decompressive craniotomy	Death	[8]
10	50	Male	I・E	Bicuspid aortic valve	Amoxicilline-clavulanate, amikacin →ceftriaxon, gentamicin	Antibiogram results	Gemella bergeri	NA	No	Survived	[9]
11	37	Male	I・E	Tricuspid valve cleft	NA	NA	Negative	Real-time PCR	Valve repair with vegetectomy	Survived	[10]
12	63	Male	I・E	Extensive dental procedure	Vancomycin, piperacillin-tazobactam, levofloxacin	No	Gram-positive cocci	Phenotypic method	No	Death	[4]
13	23	Male	I・E	Bicuspid aortic valve	Ceftriaxone, vancomycin →ampicillin, gentamicin	Antibiogram results	Gemella bergeri	NA	Mitral valve repair with vegetectomy, aortic valve replacement	Survived	[11]

*I・E* Infective endocarditis, *NA* Not applicable, *PCR* Polymerase chain reaction, *RNA* Ribonucleic acid; ^a^These cases do not mention a clinical diagnosis and further clinical data

We herein describe the first case of septic shock due to a *G. bergeri* coinfection with *E. corrodens*, which induced Lemierre’s syndrome and fulminant purpura.

## Case presentation

**Fig. 1:**
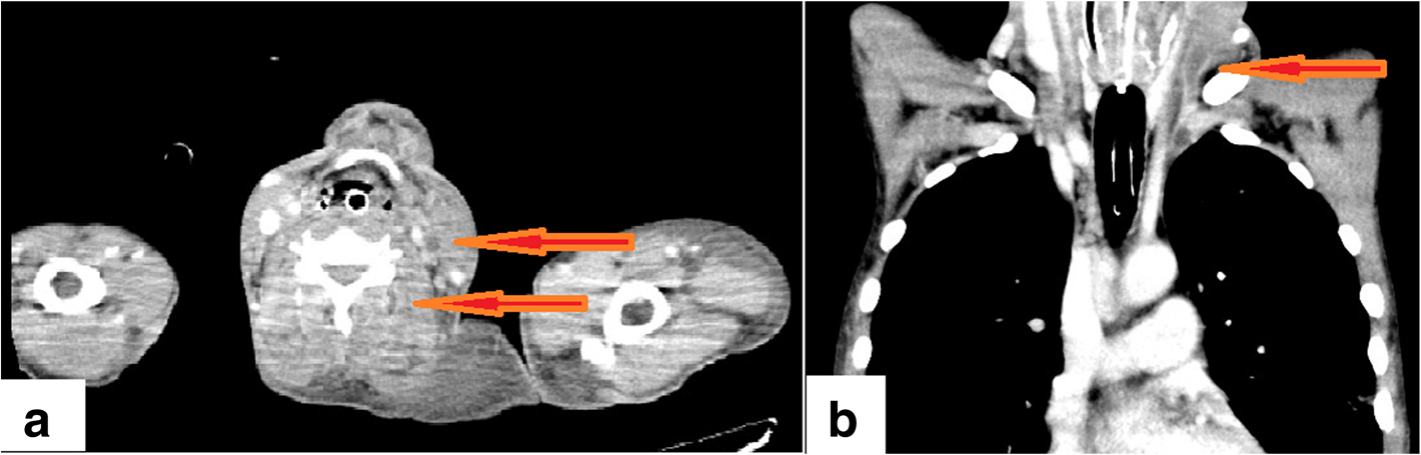
Imaging findings of a 44-year–old man who was diagnosed Lemierre’s syndrome. Contrast enhanced computed tomography on day 6 showed left internal jugular vein and vertebral vein thrombosis (arrow) (**a**. axial view, **b**. coronal view)

**Fig. 2:**
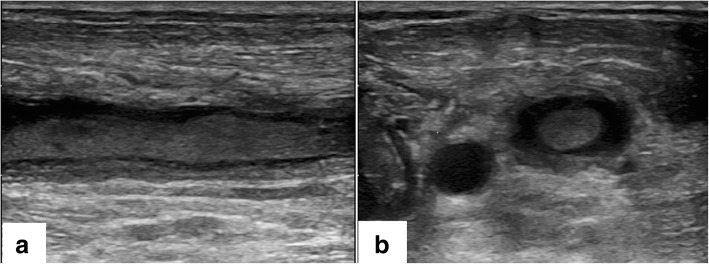
Echo scan investigation of the internal jugular vein. Echo scan of the internal jugular vein also showed the thrombosis (**a**. long axis view, **b**. short axis view)

**Fig. 3:**
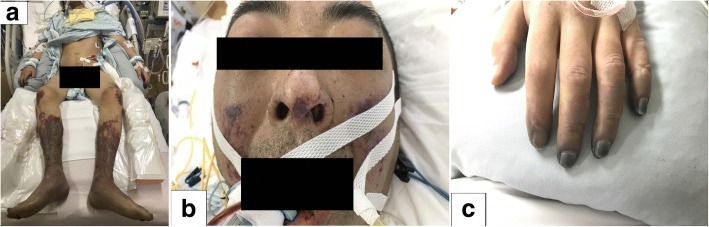
Purpura in a 44-year-old man who was diagnosed purpura fulminans. Symmetric peripheral gangrene was seen at the distal limbs, fingers, and face (**a**. bilateral limbs, **b**. buccal and nose, **c**. bilateral fingers)

A 44-year-old Asian man with a medical history of chronic sinusitis and IgG4-related ophthalmic disease who was prescribed 5 mg of oral corticosteroids (prednisolone) 2 years previously (initial dose was unknown) was transported to our hospital with dyspnea lasting for several hours. He smoked cigarettes 24 pack years but did not have a history of intravenous drug abuse, heavy drinking, or poor dental hygiene. He had not undergone dental procedures recently. On arrival, his Glasgow Coma Scale score was 11 (eye, 3; verbal, 2; motor, 6), body temperature 37.1 °C, his respiratory rate was 28/min, his blood pressure was 99/42 mmHg, and his heart rate was regular at 150 beats/min. His symptoms were not obvious because of his consciousness disturbance; his face had no skin erythema or swelling, and his neck induration could not be palpated. A purpuric eruption was covering both of legs. Transthoracic echocardiography showed a hypercontractile left ventricle without pericardial effusion, regurgitation of valves, and vegetations. Contrast enhanced computed tomography (CT) showed no obvious embolization at the bilateral pulmonary arteries, but revealed left lung ground glass opacity, and bilateral irregular lung opacities without cavitation. Fluid retention at the right maxillary sinus was also found. Laboratory test results were as follows: leukocyte count, 19,100 cells/μL; hemoglobin level, 15.7 g/dL; platelet count, 0.6 × 10^4^ cells/μL; creatinine level, 4.1 mg/dL; total bilirubin level, 3.9 mg/dL; C -reactive protein level, 45.6 mg/dL; procalcitonin level, 44.7 ng/mL; β-D-glucan level, < 6.0 pg/mL; a negative pneumococcal urinary antigen test; a negative *Legionella* urinary antigen test; prothrombin time international normalized ratio, 1.15; fibrin degradation products, 103 μg/mL; Japanese Association for Acute Medicine (JAAM) disseminated intravascular coagulation (DIC) scores, 7 points; and Sequential Organ Failure Assessment (SOFA) score, 15 points. Blood gas analysis results were as follows (10 L/minute O_2_ administered): pH, 7.174; PaCO_2_, 32.7 mmHg; PaO_2_, 177 mmHg; HCO_3_, 11.6 mmol/L; lactate 13.8 mmol/L; anion gap, 16.7 mmol/L. Owing to suspected bacterial pneumonia-induced septic shock and/or purpura fulminans, endotracheal intubation was performed, and fluid resuscitation was started immediately. After we obtained blood, sputum, and urine cultures, initial empiric antimicrobial drugs (meropenem, clindamycin, and vancomycin) were administered. Norepinephrine was initiated, titrated up to 25 μg/min. In addition, vasopressin 0.03 U/min, dobutamine 8 μg/kg/min, and hydrocortisone 200 mg/day were also added for continuous infusion. Because mean blood pressure could not be maintained at 50 mmHg despite adequate drip infusion and high dose vasopressors, venoarterial extracorporeal membrane oxygenation (VA-ECMO) was initiated due to refractory septic shock. Continuous hemodiafiltration (CHDF) was also introduced due to severe lactic acidosis, and recombinant thrombomodulin was administered for sepsis induced DIC. After the patient was admitted to the intensive care unit, his vital signs stabilized gradually. Both VA-ECMO and CHDF were tapered on day 2 post admission. Norepinephrine, vasopressin, and dobutamine were tapered on day 3, 4, and 5, respectively. Repeat contrast enhanced CT confirmed bilateral lung nodules, left internal jugular vein and vertebral vein thrombosis; following this Lemierre’s syndrome was diagnosed on day 6 (Figs 1 and 2). Although the primary focus of the infection was thought to be the right sinus, purpura worsened on both legs. He then went into shock again on day 6 (Fig. 3). Additional soft tissue infections were suspected; therefore, bilateral below the knee amputations were performed for source control. In addition to intravenous antibiotics, edoxaban (non-vitamin K antagonist oral anticoagulant) was initiated for left internal jugular and vertebral venous thrombosis. Following this, his vital signs improved without further systemic embolism. Cultures of the exudates from the skin lesions and histopathological samples did not identify any pathogens, and histopathological findings showed arterial thrombosis, and therefore, it was thought that second time shock developed due to purpura fulminans in the context of the septic shock and DIC. A tracheotomy was performed on day 13, and intravenous antibiotics and edoxaban were discontinued on day 59 with disappearance of the neck thrombosis, and bilateral lung nodules and fluid retention at the right maxillary sinus as identified on repeat CT. Lastly, he was transferred to another hospital for rehabilitation on day 121.

**Fig. 4:**
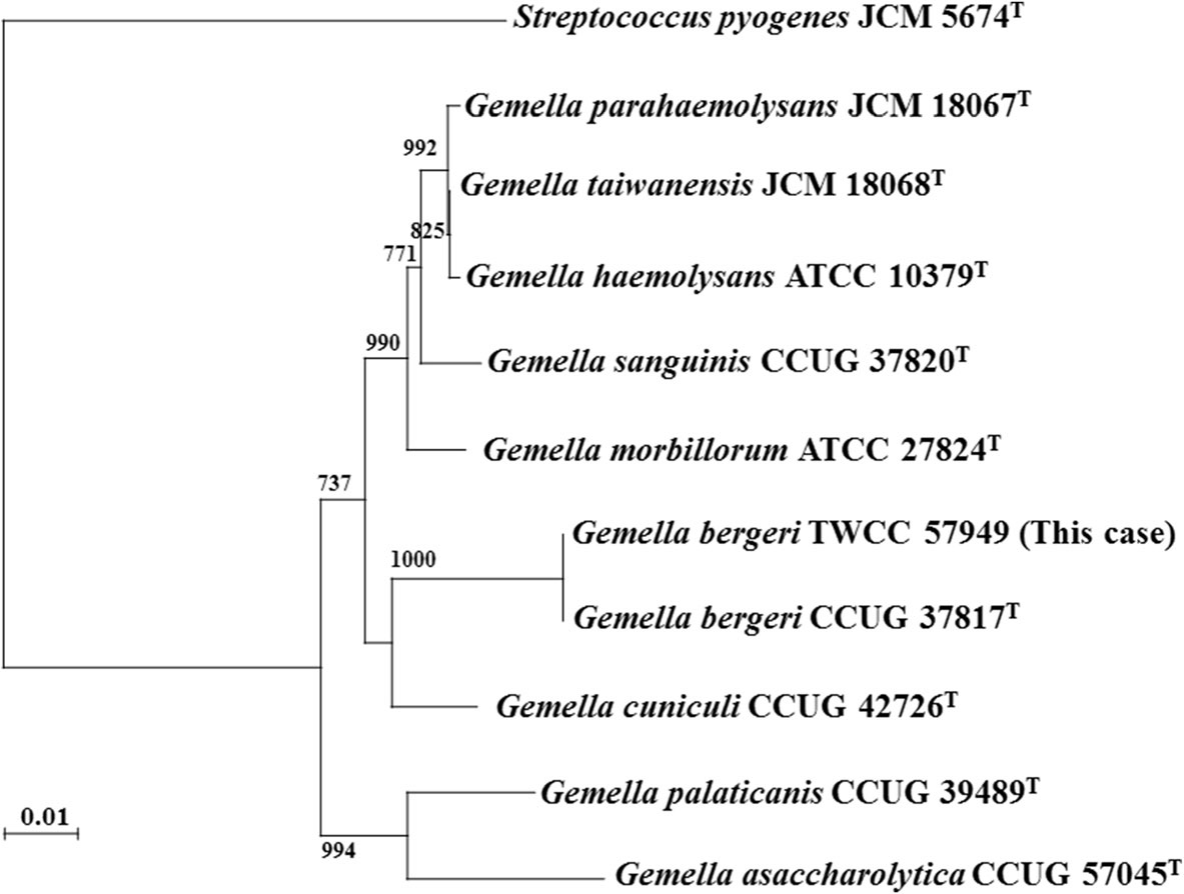
Phylogenetic tree showing the relationship among the genus *Gemella* and the strain isolated from this case. The tree was created on 16S rRNA gene sequences by the neighbor-joining method. Numbers at nodes are confidence levels expressed as percentages of occurrence in 1000 bootstrapped resampling

Gram-negative coccobacilli and gram-positive cocci were yielded from two sets of blood culture bottles (BacT/ALERT, bioMérieux, Marcy l’Etoile, France), which were taken prior to antibiotic administration. The gram-negative coccobacilli were identified as *E. corrodens* by ID Test HN-20 Rapid (Nissui Pharmaceutical Co., Ltd., Tokyo, Japan) (profile: 5220000, %ID 99.9%) and susceptibility testing was determined by Etest (bioMérieux) in Mueller-Hinton agar plates (bioMérieux) (Table 2). These gram-positive cocci were catalase-negative and showed weak-beta hemolysis on 5% sheep blood agar (Nihon Becton-Dickinson, Tokyo, Japan), initially suspected as pyogenic streptococci, after 48 h of incubation under anaerobic and 5% CO2 gas at 35 °C. This strain was not identified by the rapid ID 32 STREP system (profile 00002500000; low discrimination of *Erysipelothrix rhusiopathiae/Gemella hemolysans/Gemella morbillorum*, bioMérieux) but was identified as *G. morbillorum* by BD BBL Crystal GP (profile: 0500000100, %ID 98.5%, Becton-Dickinson, Sparks, MD, USA). It was identified as *G. bergeri* with a score value of 2.068 (species level) by matrix-assisted laser desorption ionization-time of flight mass spectrometry (MALDI-TOF MS, Autoflex II with MALDI Biotyper software ver 3.1; Bruker Daltonik GmbH, Bremen, Germany) and confirmed by 16S rRNA gene sequencing by the method described previously [12]. Phylogenetic analysis results are shown in Fig. 4. Susceptibility testing was then performed with MicroFAST Type 7 J Panels and MicroScan Walkaway-96 (Beckman Coulter, Brea, CA, USA). According to the Clinical and Laboratory Standards Institute (CLSI) document M45-A3 (Clinical and laboratory standards institute. Methods for antimicrobial dilution and disk susceptibility testing of infrequently isolated or fastidious bacteria, 3rd edition, CLSI guideline M45. Clinical and laboratory standards institute, Wayne, PA. 2015), results of minimum inhibitory concentrations of various antimicrobials and interpretation of susceptibility testing are shown in Table 2. Based on these susceptibility results and negative results of additional blood cultures on day 6, antimicrobials started empirically were changed to ampicillin-sulbactam on day 10.

**Table 2:** Susceptibility testing for *Eikenella corrodens* and *Gemella bergeri*

Pathogens and antimicrobial agents	MIC (mg/L) and interpretation of susceptibility
*E. corrodens*	
Amoxicillin-clavulanic acid	0.25/0.125 (S)
Cefotaxime	0.06 (S)
Imipenem	0.38 (S)
Clindamycin	24 (NA)
*G. bergeri*	
Benzylpenicillin	≤0.03 (S)
Cefotaxime	≤0.125 (S)
Ceftriaxone	≤0.125 (S)
Meropenem	≤0.125 (S)
Vancomycin	1 (S)
Erythromycin	≤0.125 (S)
Levofloxacin	≤0.25 (S)
Clindamycin	≤0.125 (S)

*MIC* Minimum inhibitory concentration, *NA* Not available in CLSI M45-A3 documents, *S* Susceptible

## Discussion

This is the first case of septic shock due to a *G. bergeri* coinfection with *E. corrodens.* Furthermore, this coinfection induced Lemierre’s syndrome and purpura fulminans in the patient. This case highlighted two important clinical findings. First, although *G. bergeri* and *E. corrodens* are thought to be relatively harmless microorganisms, their coinfection might become severe. Second, MALDI-TOF MS and 16S rRNA gene sequencing can identify *G. bergeri*.

*G. bergeri* was isolated for the first time by Collins et al. in 1998 [3]. Since then to our knowledge, thirteen cases have been reported [4–11] (Table 1). Three of these reports did not specifically mention a clinical diagnosis and were not described in detail. The remaining ten of them had endocarditis, eight of the ten patients showed good response to medical treatment and had a good clinical outcome, and two were fatal. In case report 9 (Table 1) the patient suffered intracerebral and subarachnoid hemorrhage secondary to rupture of a mycotic aneurysm [8], and in case 12, the patient expired due to cardiogenic shock owing to perforation of the mitral valve [4]. We obtained two sets of blood cultures twice during the treatment. *G. bergeri* and *E. corrodens* were detected in the first set of blood cultures. The second set of values on day 6 was negative, and no other pathogens were detected during the treatment. The *Gemella* and *Eikenella* were the only pathogens that were identified during the treatment; thus, these two were thought to be the causative agents associated with sepsis in our case. The coinfection of *E. corrodens* with other pathogens is common. The outcomes are apparently not severe, but sometimes become severe with certain pathogens [13]. Another study reported that both coaggregation and growth stimulation occur between *E. corrodens* and *Streptococcus* [14]. Although the virulence factors of *Gemella* and *E. corrodens* are not well understood, we recognize now that they might cause septic shock.

Early identification of the source of the infection, or the causative pathogen, and appropriate antimicrobial administration are crucial in patients with sepsis and septic shock [15]. *Gemella spp.* tend to be misidentified due to the tendency to easily decolorize in Gram staining, and commercial biochemical tests are still incapable of identifying all of the strains accurately. A molecular method such as 16S rRNA gene sequence analysis is a useful tool for accurate identification [1, 5–7]. In our case, at first, we misidentified *G. bergeri* as beta-hemolytic streptococcus spp. and changed the empiric therapy to ampicillin-sulbactam as a definitive therapy. Fortunately, the susceptibility of *G. bergeri* was good, and the patient was therefore treated adequately. However, although intravenous ceftriaxone, gentamicin and oral rifampicin are effective antibiotics [4, 6–9, 11] (Table 1), one study showed evidence of resistance to drugs such as penicillin [16], erythromycin [17], levofloxacin and aminoglycosides [1] for *Gemella.* Therefore, obtaining an accurate diagnosis of *Gemella* using a 16S rRNA gene sequence analysis should be considered if the pathogens are not identified by Gram staining or commercial biochemical tests. In addition, susceptibility testing should be performed for appropriate antimicrobial therapy.

Lemierre’s syndrome is diagnosed based on following findings: 1) primary infection in the oropharynx, 2) bacteremia demonstrated by at least one positive blood culture, 3) evidence of thrombophlebitis of the internal jugular vein, and 4) metastatic infections at one or more distant sites [18]. The overall mortality in the past 5 years based on a recent systematic review of 137 cases from 2016 was found to be 2%, and the majority of causative pathogens were *Fusobacterium*; *Gemella* were not detected [19]. Only two cases in their study that discussed infections with *Klebsiella pneumoniae* and *Streptococcus anginosus* involved fatal outcomes [19]. Anticoagulation for the internal jugular vein and ligation of the occluded vein are still controversial issues. However, if the patients failed to respond to antibiotics, they required anticoagulant treatment and/or surgical treatment to prevent further septic embolism or for resolution of septic processes [19, 20]. In our case, internal vein surgery was not needed. Evidence of further systemic septic embolism was not found, mainly due to the administration of appropriate broad-spectrum empiric antimicrobial therapy and adjunctive therapy such as anticoagulant treatment. The duration of antibiotic therapy is reported to be from 10 days to 8 weeks [19], but the reason is not explained adequately. We expect that the optimal duration will be studied further in future studies.

## Conclusion

Compromised patients such as our patient are susceptible to infection. *G. bergeri* can be a causative pathogen of septic shock. If a pathogen cannot be identified by conventional biochemical methods, MALDI-TOF MS or 16S rRNA gene sequencing should be considered.

## Abbreviations

CHDF: Continuous hemodiafiltration
CLSI: Clinical and laboratory standards institute
CT: Computed tomography
DIC: Disseminated intravascular coagulation
MALDI-TOF MS: Matrix-assisted laser desorption ionization-time of flight mass spectrometry
VA-ECMO: Venoarterial extracorporeal membrane oxygenation
